# Monitoring malaria using health facility based surveys: challenges and limitations

**DOI:** 10.1186/s12889-016-2858-7

**Published:** 2016-04-21

**Authors:** Abraham Rexford Oduro, Ernest Tei Maya, James Akazili, Frank Baiden, Kwadwo Koram, Kalifa Bojang

**Affiliations:** Navrongo Health Research Centre, P.O. Box 114, Navrongo, Ghana; School of Public Health, University of Ghana, P. O. Box LG 13, Legon, Ghana; Research and Development Division, Ghana Health Service, PMB, Accra, Ghana; Noguchi Memorial Institute of Medical Research, P. O. Box LG 581, Legon, Ghana; Medical Research Council Unit, P. O. Box 273, Banjul, The Gambia

**Keywords:** Community, Health centre, Malaria, Surveys

## Abstract

**Background:**

Health facility data are more readily accessible for operational planning and evaluation of disease control programmes. The importance, potential challenges and limitations of using facility based survey as an alternative tool for monitoring changes in local malaria epidemiology were examined.

**Methods:**

The study involved six areas within the administrative divisions of The Gambia. The areas were selected to reflect socioeconomic and malaria transmission intensities across the country. The study design involved an age stratified cross sectional surveys that were conducted during the wet season in 2008 and in the 2009 during the dry season. Participants were patients attending clinics in six health centres and the representative populations from the catchment communities of the health centres.

**Results:**

Overall participants’ characteristics were mostly not comparable in the two methodological approaches in the different seasons and settings. More females than males were enrolled (55.8 vs. 44.2 %) in all the surveys. Malaria infection was higher in the surveys in health centres than in the communities (*p* < 0.0001) and also in males than in females (OR = 1.3; *p* < 0.001). Males were less likely than females to sleep under an insecticide treated net in the communities (OR = 1.6; 95 % CI 1.3, 1.9) and in the health centres (OR = 1.3; 95 % CI 1.1, 1.5). Representativeness of the ethnic groups was better in the health centre surveys than in the community surveys when compared to the 2003 national population census in The Gambia.

**Conclusion:**

Health facility based survey though a potential tool for monitoring changes in the local epidemiology of malaria will require continuous validation of the facility and participants sociodemograhic characteristics as these may change over time. The effects of health seeking practices on service utilization and health facility surveys as an approach will also need continuous review.

## Background

Several malaria endemic countries make use of routine information available through passive health surveillance systems for programme evaluation. However, there are biases in data collection and reporting resulting from the lack of quality of service delivery, inadequate access and different health seeking behaviours [1, 2]. These factors feed into the suspicion of using passive surveillance data as a tool in routine malaria decision making [1, 2]. Current major initiatives for monitoring malaria endemicity is thus via household surveys that permit variations to be measured at the population level. Currently, the multiple indicator cluster, the malaria indicator, and the demographic and health surveys are employed [3–5]. These generate nationally representative population-based data which are comparable over time and across countries because standardized tools are employed [3–5]. One critical concern about these surveys is that most of the core indicators are for programme evaluation with few for impact assessments.

Impact indices which are often measured within these surveys include parasitaemia, anaemia and mortality in children [3–5]. As malaria incidence declines, the geographical distribution of the disease becomes patchy and national-level estimates tend to over or underestimate risk of malaria in specific regions of the country. For operational reasons, the above population-based surveys are conducted every 3 to 5 years during the dry season which is outside the peak malaria transmission period. Moreover, morbidity and mortality due to malaria is declining [6] and given that significant deaths occur at home, a definitive cause of death is not usually available. The verbal autopsy method used in these surveys is limited in sensitivity and specificity [7]. Furthermore, the surveys are restricted to children under 5 years and there is evidence of an age shift in malaria and increasingly poor correlation between fever, anaemia and malaria in many endemic countries [6, 8].

The massive deployment of life saving malaria interventions in recent years has not only resulted in a decline in disease burden but a shift in the disease pattern [6, 9]. These changes need to be monitored in order to provide up-to-date information for malaria control programmes. As no single tool would be able to monitor the changes particularly for the variety of conditions that exist in most endemic countries. A mixture of routine and real time data are necessary given the extreme resource constraints prevailing in endemic countries. Robust malaria indices at the community and facility levels are required for optimising and appropriately targeting available interventions as well as monitor spatio-temproal changes [10].

Given the challenges that are encountered using community parasite rates in monitoring low malaria transmission and the deficiencies in collection and collation of routine health facility level data [1–3], innovative approaches for characterizing malaria indices from surveys at health facility levels may be an alternative method [10]. Facility based data are more readily accessbile to aid operational planning and evaluation of control activities at the lowest administrative area [9–11]. The data provide evidence on malaria in different catchment communities which would otherwise have needed costly surveys in each of these communities. Well characterized facility based data will detect hotspots and outbreaks early enough for directed interventions to be instituted [10, 11]. The study evaluated the representativeness of malaria data from health centre-based surveys compared to that from community based surveys [10]. The paper further examines the methodological challenges and potential limitations of using health centre facility basesd survey data for routine monitoring of malaria in endemic countries.

## Methods

### Study setting

The study was carried out in The Gambia located in the western-most part of West Africa. The geography of the area is typified by sub-sahelien savannah vegetation with distinct dry and wet seasons. The mean annual rainfall ranges between 920 and 1450 mm with mean temperature ranging from 23 to 27 °C along the coast and 24 to 32 °C in other parts of the country. The landscape is dominated by the river Gambia and its flood plains. The country Gambia has an estimated population of 1.8 million and the main stay of the economy is agriculture and tourism [12–14].

In the Gambia, malaria transmission is seasonal and restricted to a solo short rainy season which typically lasts from June to October. There is a strong association between the River Gambia and malaria transmission. The alluvial bank of the river is prone to flooding and the resultant marshy vegetation and mangrove swamps generate suitable mosquito breeding sites. The important malaria vectors are *Anopheles gambiae sensu stricto, Anopheles melas* and *Anopheles arabiensis,* all members of the *Anopheles gambiae*. The dominant malaria parasite species is *P. falciparum* but *P.malariae* and *P.ovale* are also present [14, 15]. Recent data suggests that almost all infections contain *P. falciparum* species [16].

### The health system of The Gambia

This is structured into primary, secondary and tertiary levels of health care. The primary level of health care targets are settlements often with a population of few hundreds or more people. The secondary level of care is provided by major and minor health centres as used in this study as well as private clinics and is supported by a number of clinical dispensaries. Registered and enrolled nurses and other auxiliary medical staff man the health centres. Services provided by health centres include out-patient, maternal and child welfare clinics, and inpatient care but often on a smaller scale for minor health centres. Some major health centres may have resident medical doctors. Tertiary level of health care is delivered by four government hospitals and supported by the Medical Research Council (MRC) hospital [12, 13].

As far as malaria treatment is concerned all health facilities in the Gambian health care system have the capacity to diagnose malaria following the introduction of the rapid malaria diagnostic tests. Access and equity to health facilities and malaria treatment is ensured by the availability of minor health centres and community health workers who provide basic services countrywide. In addition, there are private or traditional health care providers but majority of patients first seek treatment from the formal health system and those who prefer alternative providers usually do so for cost and failure to improve after visiting the formal health system [17].

### Study design

**Fig. 1:**
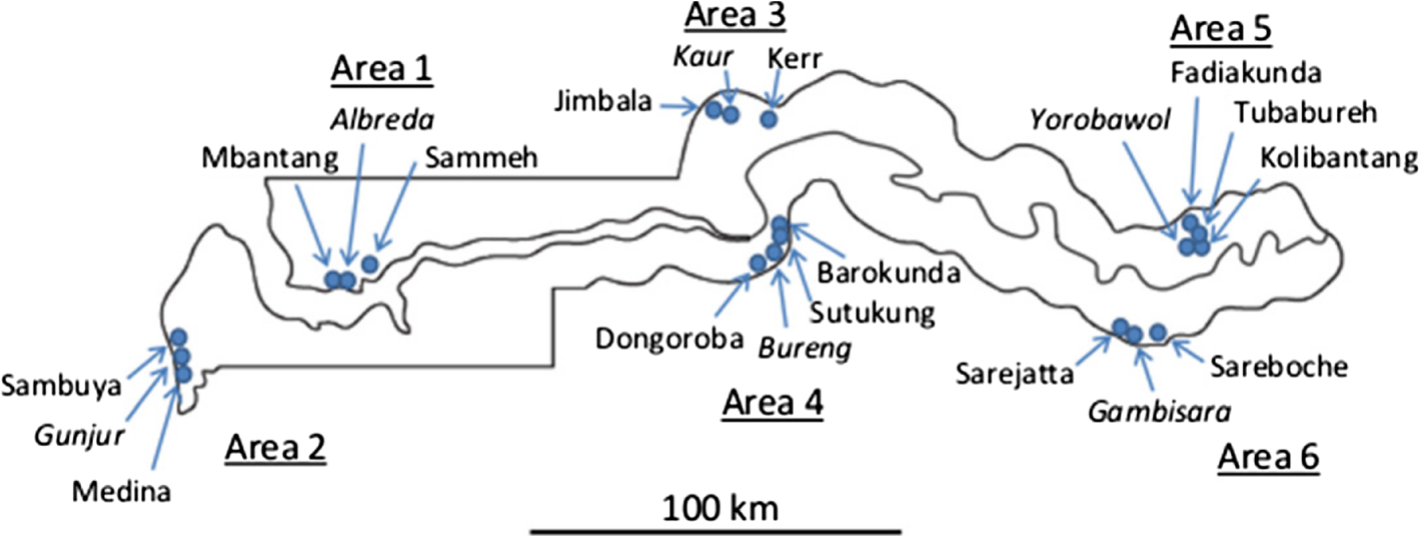
Locations of the six study areas and villages across the Gambia. They included a mix of semi-urban and rural areas and each area has a health centre *(italics)* for recruitment of participants. The map in figure 1 is from another journal, the reference [18] is to this journal

The study involved six areas within six local government districts with at least one study area selected from each of the five administrative divisions of The Gambia (Fig. 1). The sites were selected to mirror the scale of malaria transmission intensities in the country [14–16]. They included two coastal areas, two areas from mid-country and two areas from the east. For each pair of areas, one was on the north and one on the south bank of the river. They included a mix of semi-urban and rural areas, and each area had a health centre as shown in Fig. 1. The design has been previously described [10, 18].

### Study population

This included both sexes of all age groups stratified into five age categories: < 2, 2–5, 6–12, 13–25, >25 years. The inclusion of a wide range of participants’ ages was to enable variations in age pattern of malaria infection in the study area to be detected. The ages were stratified into five categories mainly for reasons of logistics and representativeness. A series of age stratified cross sectional surveys were then conducted during the wet season of 2008 (September–November) and during the dry season of 2009 (March–May). Participants were patients attending the six health centres and the representative populations from catchment villages of the health centres.

For each of the six study areas, the most centrally located health centre was used for the study for increasing representativeness. Participants’ selection criteria included all persons, both male and female living in the area. They must have been resident for a minimum of 4 weeks at the time of the survey. They or their parents/guardians (for subjects below 16 years) must have agreed to provide witnessed individual/parental informed consent, applicable assent and willingness to follow all study protocol requirements.

### Community surveys

The catchment villages of each of the health centres were listed and the Epi-Info 6 random list generator was used to randomly select two villages from each catchment area of a health centre. The village where the health centre was located was conveniently added to the other two villages for the community survey. A list of all compounds in the three villages were combined into a single list and a total of 120 compounds selected at random. Individuals in the compounds were asked to converge at a predetermined location on survey days. All participants who consented were enrolled consecutively into the study according to their age groups until the required number was obtained for each agegroup. For each participant enrolled, a study questionnaire was administered to collect basic information on sociodemographic, clinical and socioeconomic characteristics. The same villages and study procedures were used for both the wet and dry season surveys. To ensure that data from the community surveys represented the spectrum of malaria transmission in The Gambia, sites were selected from both banks of the River Gambia, the coastal areas , and the middle and eastern part of the country.

### Health centre surveys

All patients attending the selected health centres during the study period were eligible and were invited to participate regardless of their clinical presentation. Those from whom voluntary informed consent was obtained were enrolled consecutively into the study according to their age groups until the required number per age group per health centre was achieved. The entire data were collected concurrently with the community surveys. A structured questionnaire was administered to collect study information from each participant at the health centres. The questionnaire captured information on socio-demographic characteristics, current signs and symptoms and anti-malarial measures. To ensure that data from the health centre surveys represented that from the catchment communities and the spectrum of malaria transmission in The Gambia, continuous enrolment of participants into health centre surveys was adopted.

### Sample size estimation

This was based on previous malaria prevalence estimates. For a given prevalence estimate p and precision d, the 95 % confidence interval around p is p ± d, where d = 1.96x√ (p (1-p)/n). We evaluated n for a range of *p* values to give a suitable value of d. Different sample sizes with precision ranging from d = 0.05 (5 %) to 0.1 (10.0 %) for prevalence estimates of 12, 25, 30, 35 and 61 % that included the two extremes of parasite prevalence estimates (12 & 61 %) reported earlier in The Gambia [16, 19]. We took into consideration the range of parasite prevalence documented in different age groups at different times over recent years in The Gambia. We assumed 25 % to be the average current parasite prevalence in children less than 6 years of age . Therefore, a sample size of 120 per age group per area would provide over 80 % power at a 5 % level of significance to detect an 18 % difference in parasite prevalence (risk ratio =1.72) between any two age groups or between study sites. It would also provide estimates to within +/- 8.0 % of the true value. The total sample size per area or per health centre was calculated to be 600 (120 x 5 age-groups) people.

### Data and statistical analysis

All study data were captured using standard study forms designed specifically for this study. Only designated, trained study staff completed these forms. All completed forms were checked for internal consistency and all queries were resolved routinely in the field. Data were double entered and validated in OpenClinica database which is Good Clinical Practice (GCP) compliant. The completed datasets were verified and cleaned. Initial analyses checked logical inconsistencies, data completeness and quality. All statistical analyses and estimates were computed using STATA (2012 StataCorp) software according to a predefined analytical plan. All statistical analysis, estimations and hypotheses testing were based on parametric methods and were two sided with statistical significance level set at *p*-value of ≤ 0.05.

### Quality control procedures

All participants were enrolled consecutively according to their age groups until the required number was obtained to ensure representativeness. The same villages, health centres and study procedures were used for the surveys in the two seasons. Both the qualitative and quantitative discrepancies of the malaria parasite results were reviewed by a senior microscopist who was not associated with the study. In addition, the senior microscopist read 10 % randomly selected negative slides. For the haemoglobin test, proper training and handling of the equipment, and regular calibrations and standardization of the Hemocue® Photometer were ensured. The duplicate optical density (OD) of the ELISA results were averaged and normalised against a positive control. The cut-off for sero-positivity was mean plus three standard deviations (Mean +3SD) of the non-immune controls. All data management processes followed the standard operating procedures of the data management department of the Medical Research Council Unit in The Gambia.

### Ethical consideration

The scientific justification for this protocol was the need to document the current malaria situation in The Gambia and to help to identify the strengths and weaknesses of community and health facility approaches to malaria data collection. The Gambia Government and the Medical Research Council Unit Joint Ethics Committee gave approval to this research protocol.

## Results

**Fig. 2:**
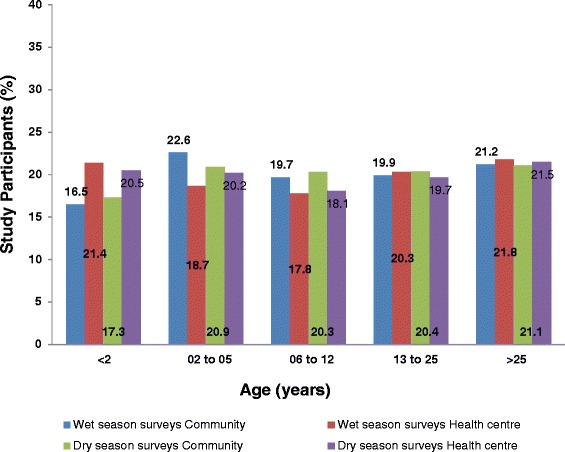
Proportions of the total study participants recruited by agegroup, surveytype and the season of malaria transmission. Colours represent season and surveytype and the bars represent the percentages of the recruited partcipants

A total of 16,230 participants were enrolled, 53.3 % from the health centres and 51.8 % during the wet season. Each of the four arms of the study achieved the required total sample size of 3600 people and the five age groups recruited suficient numbers per study arm (Fig. 2). Participants less than 2 years of age had relatively low numbers of recruitment overall - 16.5 % in the wet season and 17.3 % in the dry season in the community surveys. This under 2 years age group had the narrowest class interval and it was therefore difficult to recruit the targeted numbers. In the health centre surveys, the 6 to 12 years age group had the lowest proportions in the wet season (17.8 %) and in the dry season (17.9 %) surveys (Fig. 2).

Sociodemographic characteristics of participants were not always comparable between the community and health centre surveys particulary for gender and ethnicity. More females than males were recruited in the community and health centre surveys (Table 1). More females were enrolled in the health centres than in the community surveys in the wet season (58.0 % vs. 53.4 %; *p* < 0.05) and in the dry season (58.4 % vs. 52.7 %; *p* < 0.05). Mandinka (47.2 %), Fula (29.2 %) and Wollof (9.3 %) were the major ethnic groups recruited into the study. Health centre participants were more likely to be married and participants in the community surveys more likely to be farmers Table 1. Majority of the participants were not married, had no formal education and occupation (Table 1).

**Table 1:** Background and socio-demographic attributes of the study participants

Variables		Wet season, *n* (%)			Dry season, *n* (%)		
		Community	Facility	*p*-value	Community	Facility	*p*-value
		*N* = 3870	*N* = 4539		*N* = 3707	*N* = 4098	
Sex	Females	2063 (53.4)	2637 (58.0)	<0.001	1956 (52.7)	2369 (58.4)	<0.001
Ethnicity	Mandinka	2243 (58.1)	1723 (37.9)	<0.001	2057 (55.4)	1615 (39.6)	<0.001
	Fula	996 (25.8)	1454 (32.0)	<0.001	1023 (27.6)	1256 (30.8)	0.002
	Wollofs	295 (7.6)	516 (11.4)	<0.001	276 (7.4)	413 (10.1)	<0.001
	Others	326 (8.5)	849 (18.7)	<0.001	355 (9.6)	793 (19.5)	<0.001
Marital status	Married	1010 (26.2)	1272 (33.8)	<0.001	928 (25.0)	1204 (29.5)	<0.001
	Single	177 (4.6)	158 (4.2)	0.372	178 (4.8)	153 (3.8)	0.029
	Widowed	60 (1.5)	90 (2.4)	0.003	38 (1.0)	79 (1.9)	0.001
	Not applicable	2613 (67.6)	2242 (59.6)	<0.001	2563 (69.1)	2640 (64.8)	<0.001
Education	None	1774 (46.0)	2648 (58.4)	<0.001	1642 (44.4)	2336 (57.4)	<0.001
	Arabic	1207 (31.3)	1176 (25.9)	<0.001	1174 (31.7)	978 (24.0)	<0.001
	Primary	587 (15.2)	407 (9.0)	<0.001	614 (16.6)	489 (12.0)	<0.001
	Secondary	287 (7.5)	306 (6.7)	0.154	270 (7.3)	269 (6.6)	0.224
Occupation	None	2564 (66.9)	2960 (65.3)	0.123	2491 (68.3)	2605 (64.5)	<0.001
	Farming	774 (20.2)	655 (14.4)	<0.001	614 (16.8)	373 (9.2)	<0.001
	Trading	46 (1.2)	91 (2.0)	0.004	54 (1.5)	84 (2.1)	0.047
	Civil service	36 (0.9)	57 (1.3)	0.082	24 (0.6)	53 (1.3)	0.002
	Housewife	173 (4.5)	583 (12.9)	<0.001	265 (7.3)	246 (6.1)	0.034
	Others	241 (6.3)	188 (4.1)	<0.001	201 (5.5)	675 (16.7)	<0.001

Overall prevalence of malaria slide positivity was 24.0 % in the health centres and 12.4 % in community surveys in the wet season (OR = 2.2; 95 % CI 1.9, 2.5). In contrast, prevalence of malaria was higher in the community (2.2 %) than in the health centre surveys (1.1 %) in the dry season (OR = 1.9; 95 % CI 1.3, 2.7) Table 2. In the community surveys, prevalence on the south bank was 15.9 % compared with 8.8 % on the north bank in the wet season (OR = 2.0; 95 % CI 1.6, 2.4). In the health centre surveys, prevalence was 30.0 % on the south bank compared with 17.2 % on the north bank in the wet season (OR = 2.1; 95 % CI 1.7, 2.3). Similar findings were observed in the community and in the health centre surveys in the dry season. The prevalence of gametocytes was low and similar in the community (1.5 %) and in the health centre (1.4 %) surveys in the wet season (OR = 1.0; 95 % CI 0.7, 1.4). In both community and health centre surveys parasite prevalence increased with age rising from year one to a peak in 11–15 year olds (early adolescence) and thereafter declined. In all age groups, malaria prevalence was higher in the health centres than in the community surveys except the differences did not reach statistical significance in those less than 1 year and greater than 25 years.

**Table 2:** Comparaison of attributes of malaria indices by season and survey type

Attributes	Wet season surveys			Dry season surveys		
	Community	Facility	*p*-values	Community	Facility	*p*-values
Number (*N*)	3870	4539		3707	4098	
Asexual parasites, *n* (%)	478 (12.4)	1088 (24.0)	<0.001	80 (2.2)	46 (1.1)	<0.001
Geomean parasite density (μl)	217	12,206		337	280	
Parasitaemia ≥1000 μl, *n* (%)	111 (2.9)	914 (20.1)	<0.001	20 (0.5)	17 (0.4)	0.509
Sexual parasites, *n* (%)	56 (1.45)	65 (1.43)	0.939	7 (0.2)	4 (0.1)	0.250
Anti MSP1_19_ (*N*)	3517	3385		3388	3365	
Sero-positive, *n* (%)	736 (20.9)	1122 (33.2)	<0.001	712 (21.0)	696 (20.7)	0.762
Temperature (°C) (*N*)	3860	4497		3707	4069	
Mean (SD)	36.8 (0.5)	37.3 (1.0)	<0.001	36.7 (0.4)	36.9 (0.8)	<0.001
Fever (≥37.5 °C), *n* (%)	214 (5.5)	1410 (31.4)	<0.001	83 (2.2)	782 (19.2)	<0.001
Haemoglobin (g/dl) (*N*)	3821	4407		3709	3970	
Mean (SD)	11.1 (2.0)	11.0 (2.7)	0.060	11.6 (1.8)	11.0 (2.1)	<0.001
Anaemia (Hb ≤8), *n* (%)	283 (7.4)	440 (10.0)	<0.001	127 (3.4)	317 (8.0)	<0001

The mean haemoglobin concentration in the community (11.05 g/dl) and health centre (11.02 g/dl) surveys were similar in the wet season (*P* > 0.05) but was higher in the community (11.61 g/dl) than in the health centre (11.01 g/dl) surveys in the dry season (*P* < 0.001) Table 2. In the community surveys there was significant seasonal variation in anaemia in the east of the country but health centre surveys had significant seasonal variation in the middle part of the country. Overall, males were less likely than females to sleep under a mosquito net in the community surveys (self reported) (OR = 1.6; 95 % CI 1.3, 1.9) and in the health centre surveys (OR = 1.3; 95 % CI 1.1, 1.5) in the wet season. Adolescent males were less likely than adolescent females to sleep under a mosquito net in the community (OR = 3.1, 95 % CI 2.1, 4.4) and in health centre surveys (OR = 2.4; 95 % CI 1.7, 3.1). Prevalence of malaria was higher among non-Insecticide Treated Net (ITN) users compared with those using ITN in the community (18 vs.12 %; OR = 1.6; 95 % CI 1.3, 2.1) and in the health centre surveys (31 vs. 22 %; OR = 1.6; 95 % CI 1.3, 1.8).

## Discussion

Given the challenges that are likely to be encountered monitoring malaria using community based surveys in low transmission settings [9, 16] and routine health facility malaria data [1–3]. Well characterized data from facility based malaria surveys are more likely to provide information on malaria in different communities which would otherwise have required several costly surveys in each of these communities [10, 11]. However, sociodemographic characteristics and malariometric indices from a health centre-based survey should be similar to those obtained from the surrounding catchment communities in order for such a tool to be effective for monitoring the changes that are occur in malaria transmission. Different health seeking practices and lack of representativeness in the data are some of the challenges that facility based survey as a monitoring tool is likely to encounter. To mitigate these, a tool of this nature should ensure continuous enrolment in order to capture patients of all shades including early and late health care seekers as well as those close to and far away from health facilities.

This study was designed to ensure a representative sample and to enable in-depth assessment of health facility data in relation to data from the local catchment communities. The results revealed however that overall characteristics of the participants in the two methodological approaches were in most cases significantly different. The findings showed that more females than males were enrolled and this was particularly the case in the health centre surveys irrespective of the season of malaria transmission. This maybe due to the fact that in general, women have higher utilization of medical services and outpatient attendance than men [20–22]. It could also be because women’s access to preventive and therapeutic measures tend to be lower than men or more prone to illnesses compared to their male counterparts [21, 22].

Women are also more likely than men to use health facilities as part of motherhood activities; sending children to welfare clinics or accompanying family members to seek health care, and are thus more likely to report sick themselves. Over representation of women in facility based surveys have implications for the interpretation of health facility based survey data. If women have a different risk of malaria infection than men, then recruiting more women will affect the overall estimates of malaria indices in the health centre surveys compared to that in the catchment communities and this may lead to wrong interpretation and policy initiatives. The overall risk of malaria in the study, adjusting for age did not vary by gender despite the fact that less men, particularly the adolescents, were less likely to sleep under insecticide treated bednet. Nevertheless, the uneven balance of power, financial resources and inequitable access to health care as a result of gender and other social roles means women may be more vulnerable to diseases including malaria [21–23].

The major ethnic groups in The Gambia are the Mandinkas, the Fullas and the Wollofs [12]. In this study, more Mandinkas, the largest ethnic group were recruited in the community surveys while the fulla and other minorities were more in the health centre surveys. The Wollof were statistically equal in both surveys. Within surveys however, the ethnic groups were better represented in the health facility than in the community surveys when compared to data from the last population census in The Gambia [12]. The possible explanation of this ethnic differences may be due to the fact that siting of health facilities are not based on ethnicity but settlements tend to be based on ethnicity. Some of the villages tend to be occupied predominantly by one ethnic group. The implication is that if a village for the community survey was selected at random and the residents were predominantly of one ethnicity, then all participants were likely to be from that ethnic group. This situation does not arise with health centre surveys. The significance of ethnic imbalance is that it may have undermined the representativeness of data if there were significant ethnic differences in the risk of malaria. It has previously been shown that the Mandinkas in the Gambia are less at risk of malaria infection and disease than other major ethnic groups. This had been explained that the Mandinkas had historically been more likely to use mosquito nets even for other reasons than the other ethnic groups [24, 25]. Simple random sampling of households increases representativeness of community surveys but is associated with high cost and complexities and could not be used in this study.

Recent evidence indicates an age shift in the epidemiology of malaria [7, 9], and this informed the inclusion of participants across all ages to enable variations in age pattern of malaria to be described. However, recruiting children 6-12 years was more difficult in the health centre than in the community surveys because a few of them came to the health facility to seek medical attention. As the only age group in the study in which all participants are of school going age, this may affect their utilization of health facilities. Recruitment of a representative sample of school children would have prolonged the duration of health centre surveys. Such a decision would increase the cost of health centre survey methods for monitoring malaria as recent evidence suggest a higher risk of malaria infection in school children. The increase in malaria parasite prevalence in older children suggests that school based surveys could be a useful supplementary approach for monitoring malaria in older children. Since the study showed that young adolescents are less likely to use ITNs compared to other age groups [10], targeting this age group in schools for health education may increase their use of ITNs.

In general, seeking health care is determined by sociobiological factors such as age, sex, ethnicity, occupation, educational level, among others. Health seeking habits influence utilization of health services and interventions. The decision to engage with a particular health facility is determined by several factors including socio- cultural, cost of care and access. Others include distance, physical access, type of illness and quality of care from a particular source [20]. Health seeking practices can influence measured malaria indices in either surveys. Increased health seeking behaviour will lead to low *P. falciparum parasite* rate estimates in community surveys while decrease in health seeking behaviour may lead to potential biases in indices measured in health facilities. As a potential tool for monitoring changes in the local epidemiology, health facility based surveys will require complete documentation of the effects of health seeking practices as part of the validation process.

Limitations of the study: The results may be interpreted with caution because the data from a research study setup may not exactly reflect what actually happens in normal health facility settings. Several factors may account for this including available resources, health care provision including diagnostic abilities, malaria care among others. These may affect reliability and hence importance of such health facility survey data. In addition, other contextual issues related to the health system vary between facilities and this may further affect the reliability of health facility data.

There may be inadequate representation of participants from the selected villages in the health facility surveys data as the number of villages per health centre were too few to give a representative sample from the catchment area. Diversity in parasite prevalence within an area as well as heterogeneous ethnic groups, and varying patterns of health seeking behaviour makes sample representativeness key in monitoring malaria. The cross-sectional design also limited seasonal analysis and our ability to assess trends in the indices.

## Conclusions

Health centre surveys have the potential to detect and respond to hotspots of malaria transmission, infection and disease burden. There is however the need to examine the effects of health centre and particpants characteristics on the representativeness of health centre survey data. Health facility surveys may be useful surveillance tools for evaluating area specific malaria control activities and for monitoring changes in the local malaria epidemiology. The potential for using such a surveillance tool could be improved by continuous validation of health centre data and the effect of health seeking practices on the measured malaria indices.
